# Discrimination of secondary hypsarrhythmias to Zika virus congenital syndrome and west syndrome based on joint moments and entropy measurements

**DOI:** 10.1038/s41598-022-11395-2

**Published:** 2022-05-05

**Authors:** Priscila Lima Rocha, Washington Luis Santos Silva, Patrícia da Silva Sousa, Antônio Augusto Moura da Silva, Allan Kardec Barros

**Affiliations:** 1 Department of Electrical Engineering, Laboratory for Biological Information Processing (PIB), Federal University of Maranhão (UFMA), São Luís, MA CEP 65080-805 Brazil; 2 Department of ElectroElectronics, Federal Institute of Maranhão (IFMA), São Luís, MA 65030-005 Brazil; 3 Department of Medicine, University Hospital of the Federal University of Maranhão, São Luís, MA 65080-805 Brazil; 4 Department of Public Health, Federal University of Maranhão, São Luís, MA 65080-805 Brasil

**Keywords:** Data processing, Machine learning, Predictive medicine

## Abstract

Hypsarrhythmia is a specific chaotic morphology, present in the interictal period of the electroencephalogram (EEG) signal in patients with West Syndrome (WS), a severe form of childhood epilepsy and that, recently, was also identified in the examinations of patients with Zika Virus Congenital Syndrome (ZVCS). This innovative work proposes the development of a computational methodology for analysis and differentiation, based on the time-frequency domain, between the chaotic pattern of WS and ZVCS hypsarrhythmia. The EEG signal time-frequency analysis is carried out from the Continuous Wavelet Transform (CWT). Four joint moments—joint mean—$\mu _{(t,f)}$, joint variance—$\sigma _{(t,f)}^2$, joint skewness—$\lambda _{(t,f)}$, and joint kurtosis—$\kappa _{(t,f)}$—and four entropy measurements—Shannon, Log Energy, Norm, and Sure—are obtained from the CWT to compose the representative feature vector of the EEG hypsarrhythmic signals under analysis. The performance of eight classical types of machine learning algorithms are verified in classification using the k-fold cross validation and leave-one-patient-out cross validation methods. Discrimination results provided 78.08% accuracy, 85.55% sensitivity, 73.21% specificity, and AUC = 0.89 for the ANN classifier.

## Introduction

The use of engineering tools, such as signal processing and machine learning techniques to pattern recognition, has made great advancements in the biomedical area. The modern equipment used to perform tests on patients generate a large amount of biological signals. These signals can be processed and analyzed by algorithms that provide robust answers to assist health professionals in diagnosing and treating diseases in a fast and reliable way^1^

Among the several physiological sources of signals, the brain stands out, as a human body organ widely studied nowadays. Therefore, the capture of the brain’s electrical activity is done through the Electroencephalogram (EEG) exam, in which electrodes are positioned on the scalp, the cerebral cortex or the brain of the individual. These activities are transmitted through the electrodes to amplifiers, and then pass through filters that regulate the frequency to be registered^2,3^.

Epilepsy, the main disease studied through EEG, is a disease of the brain characterized by spontaneous and recurrent seizures, and may be the result of paroxysmal, excessive, and synchronous discharges from a neuronal population. Many factors may be involved in these abnormal discharges, and the clinical manifestations depend on the type and location of the neuronal group involved. Thus, several researches based on EEG signals seek to understand the functioning of neural circuits involved in epileptic seizures. This way, they offer solutions for prediction and classification of seizures and epileptic syndromes, thus generating a strong social impact^4,5^.

Epilepsy can start in the first months of the individual’s life, receiving special classifications according to its characteristics at this stage of the child’s development^6,7^. Among the epilepsies of childhood, there is West Syndrome, a rare and severe form of epilepsy whose etiology (cause and origin) is still not defined. West Syndrome was one of the first epileptic syndromes described, studied by the English physician William James West, who detected the symptoms in his son^8^. This epilepsy in childhood is characterized by the triad: the presence of spasms, delayed cognitive development, and the hypsarrhythmia pattern on the electroencephalogram examination^9,10^. Spasms are the clinical signs that the patient expresses during seizures. They are presented as myoclonic–tonic seizures (spasms) that can be characterized by flexor, extensor, or mixed movements. Evidence of delayed or regressed psychomotor development can be obtained from the patient’s history and physical examination, although children who are brought to the doctor soon after the onset of spasms may still show good development^11,12^. Unlike these two symptoms that are noticeable to caregivers, there is hypsarrhythmia, which is only detected by acquiring the brain signals, i.e., the EEG.

Hypsarrhythmia is a chaotic rhythm pattern, present in intervals between seizures, characterized by slow waves of high amplitude, mixed by peaks and discharges of sharp waves, also of high amplitude, without phase concordance, that vary in topography and duration^13,14^. This variability is probably responsible, at least in part, for the poor reliability among healthcare professionals when classifying hypsarrhythmia. However, identification of this pattern is still considered an important clinical biomarker of poor prognosis. There is even greater controversy concerning the specific definitions relating to the modified hypsarrhythmia patterns^15,16^.

Due to the importance in the identification of hypsarrhythmia for the adequate treatment of patients with West Syndrome, some works have been proposed with this purpose, using digital signal processing and machine learning techniques^17–19^.

However, recently, research efforts in EEG signal processing have been focused to propose improvements in the quality of life of children born with microcephaly caused by the Zika virus (ZIKV). In 2015, an outbreak of the Zika virus infection was reported in the Northeast of Brazil, which was subsequently associated with an increase in neonates born with microcephaly^20,21^. Due to the severe central nervous system involvement, it was observed that children with Zika Virus Congenital Syndrome (ZVCS) presented seizures as soon as in the neonatal period, and with an increase in the frequency of these seizures during development, being the occurrence of epileptic seizures more evident from three months of age and the epileptic spasms the most common type^22,23^.

From the EEG examinations performed in infants with seizures, the presence of the electroencephalographic pattern of hypsarrhythmia was also observed. In this specific syndrome, the identification of hypsarrhythmia becomes more challenging because the EEG signal presents several abnormalities^24,25^. Thus, there is the importance of hypsarrhythmia detection in these patients. But, there is a lack of applications in EEG’s of children with microcephaly caused by the Zika virus. Reference^26^ proposed in his thesis a signal decomposition methodology into small waves able to assist in the identification of the hypsarrhythmia electroencephalographic pattern in children with Zika Virus Congenital Syndrome.

Although hypsarrhythmia has been studied and known by neurologists since 1841, through the description of West Syndrome, the hypsarrhythmia characterization in infants with the Zika virus microcephaly are still very superficial. Then, the question arises whether there is a difference between the hypsarrhythmic pattern that occurs in those born with ZVCS and those from West Syndrome. Since the emergence of ZVCS microcephaly cases, many questions about the characterization of this disease are still open, among them, determining whether the hypsarrhythmia in ZVCS follows the same electroencephalographic pattern as the West Syndrome hypsarrhythmia.

The initial treatments for seizure control and attenuation of the chaotic interictal pattern in children with microcephaly ZVCS are based on those for patients with West Syndrome^27^. However, given the hypothesis that hypsarrhythmia has distinct characteristics in these two syndromes, since the etiology of this pattern is diverse and poorly known, healthcare professionals can more effectively target treatments for ZVCS patients. In addition to providing a better prognosis for these children, the description of hypsarrhythmia in microcephaly caused by the Zika virus relating to West Syndrome hypsarrhythmia is a great contribution to research concerning Zika Virus Congenital Syndrome.

The main contributions of this paper can be summarized as follows: (1) Original approach on characterization and discrimination of the hypsarrhythmia pattern, present in the EEG signal in WS and ZVCS; (2) CWT reveals the peculiarities of the energy distribution profile of the EEG hypsarrhythmic segments in the time-frequency domain; (3) Only eight features extracted from CWT-four joint moments and four entropy measurements—represent the hypsarrhythmia pattern segments with ZVCS and WS; (4) ANN reached the best classification results reliably revealing the differences between the hypssarithmia pattern in the two syndromes validated through the leave-one-patient-out approach; (5) the results demonstrate an opportunity for new researches related to the specificities of ZVCS, such as the hypsarrhythmia pattern, where new medications and more effective treatments can be proposed in order to improve the quality of life of children with ZVCS.

Considering the above, this paper proposes the development of a computational methodology for the analysis and discrimination of the hypsarrhythmia pattern given in microcephaly caused by the Zika virus and the hypsarrhythmia pattern arising from West Syndrome. We extracted joint moments and entropy measurements from the time-frequency energy distribution obtained from the Continuous Wavelet Transform. So, these joint moments and entropy measurements were used as inputs of state-of-the-art machine learning methods that were able to differentiate the two EEG hypsarrhythmia patterns.

## Materials and methods

**Figure 1:**
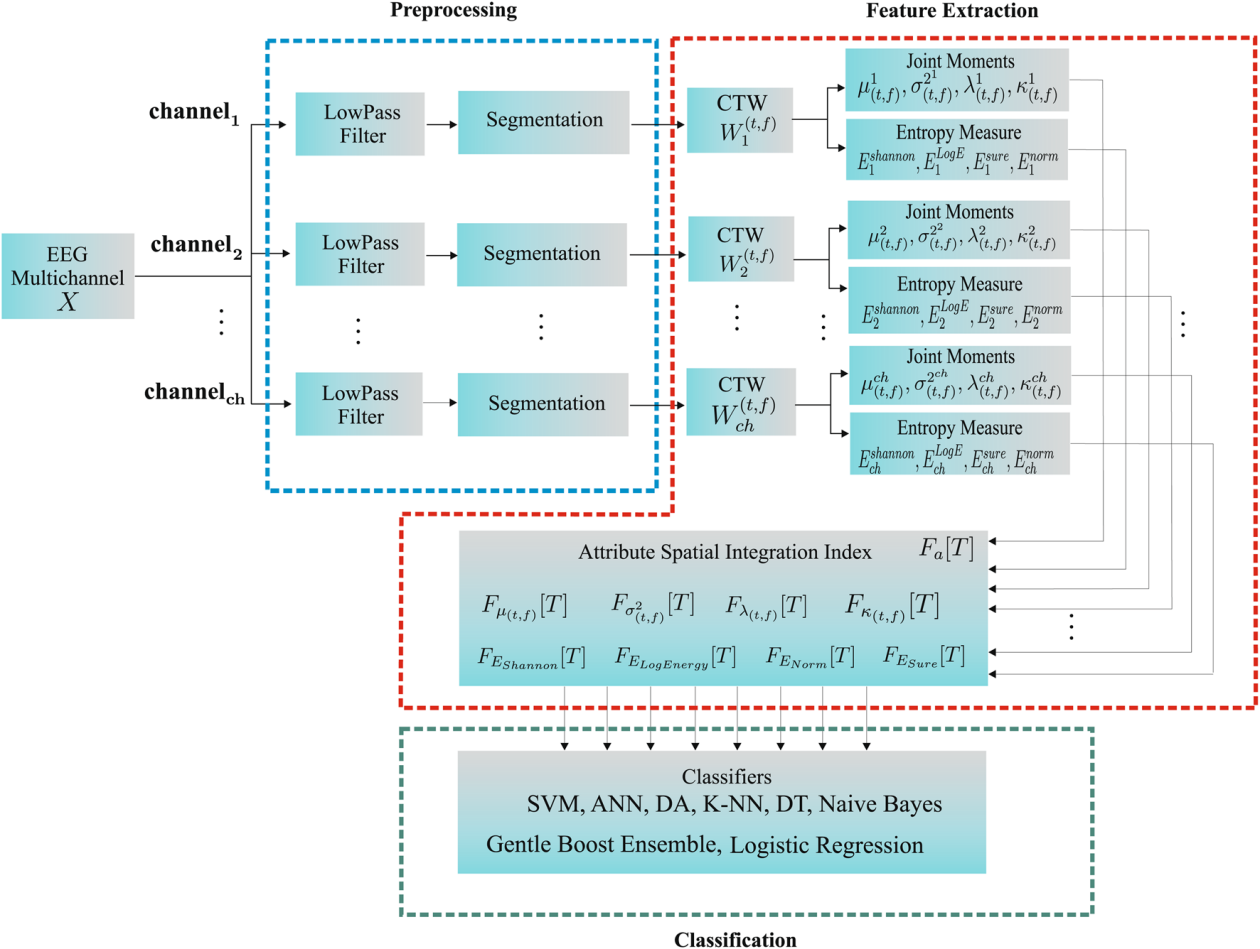
Flow chart of the proposed methodology.

In this work, each EEG signal channel was preprocessed, segmented, and the energy distribution in the different frequencies over time was determined from the Continuous Wavelet Transform, including the principal subbands (delta ($\delta$ 0–4 Hz), theta ($\theta$ 4–8 Hz), alpha ($\alpha$ 8–16Hz), beta ($\beta$ 16–32 Hz), and gamma ($\gamma>$ 32 Hz). Then, the hypsarrhythmia segments of the interictal EEG signals of Zika Virus Congenital Syndrome (Hips-ZVCS) patients are differentiated from the hypsarrhythmia segments of the interictal EEG signals of West Syndrome (Hips-WS) patients by extracting features from the time-frequency energy distribution generated by the CWT. Thus, the feature vector is composed by the first four joint moments—joint mean ($\mu _{(t,f)}$), joint variance ($\sigma _{(t,f)}^2$), joint skewness ($\lambda _{(t,f)}$), and join kurtosis ($\kappa _{(t,f)}$), and four entropy measurements—Shannon’s Entropy, Log Energy Entropy, Sure Entropy, and Norm Entropy. At the end of the feature extraction stage, each measurement obtained from each channel of an EEG segment is combined into a single index through an approach called attribute-level spatial integration. The integration indices make up the feature vector that represents the Hips-ZVSC and Hips-WS classes. The statistical significance of the indices of joint moments and the indices of the entropy measurements were done through the Kolmogorov–Smirnov and Mann–Whitney tests. Analysis of the individual relevance of each index as well as the relevance of the index set for maximum separability between the Hips-ZVSC and Hips-WS segments is performed. This study is conducted to verify the possibility of reducing the dimensionality of the feature vector. Finally, these indices are used as input vectors for the eight most common machine learning techniques: SVM, Discriminant Analysis, K-NN, Gentle Boost Ensemble, Decision Tree, Logistic Regression, Naive Bayes, and Artificial Neural Network classifiers. Then, we obtained the results of accuracy, sensitivity, and specificity, besides the ROC curve, the area under the ROC curve (AUC), the Cohen’s kappa coefficient ($\kappa$), and the Matthews correlation coefficient (MCC) of the classification of EEG segments with hypsarrhythmia from the ZVCS patients and the EEG segments with hypsarrhythmia from the WS patients using k-fold cross validation and leave-one-patient-out cross validation as validation methods. All procedures of EEG signal processing were written in MATLAB R2019b. The block diagram of the proposed methodology is shown in Fig. 1.

### EEG signals database

The hypsarrhythmia patterns for the proposed analysis came from EEG signals collected from patients with Congenital Zika Virus Congenital Syndrome and patients with West Syndrome, being treated at *Casa de Apoio Ninar*, located in São Luís, Maranhão, Brazil. Casa de Apoio Ninar, along with Centro de Referência em Neurodesenvolvimento, Assistência e Reabilitação de Crianças do Complexo Hospitalar Dr. Juvêncio Matos is a support center for children with neurological diseases, including microcephaly caused by the Zika virus in the state of Maranhão. The first set of EEG signals is comprised of examinations from 30 children with microcephaly caused by the Zika vírus. Among these children, 13 patients show interictal traces of the hypsarrhythmia pattern. There are 8 boys and 5 girls, whose ages range in 3.62 ± 1.2 months and whose cephalic perimeters during birth were 29.5 ± 2.46 cm. All infants studied met clinical criteria for congenital Zika syndrome, according to the World Health Organization recommendations. Examinations of 13 children diagnosed with West Syndrome make up the second signal set. Among these children, there are also 8 boys and 5 girls, whose ages ranged from 9.38 ± 1.32 months. We did not have access to the information on a likely etiology of WS in the patients. These signals were sampled at a frequency of $f_a = 128$ Hz and recorded with Neuromap software. The electrodes were placed on the scalp of the children during spontaneous sleep, following the International 10/20 System, in which the combination of these electrodes generated 24 channels: ‘Fp1-F3’, ‘F3-C3’, ‘C3-P3’, ‘P3-O1’, ‘Fp1-F7’, ‘F7-T3’, ‘T3-T5’, ‘T5-O1’, ‘Fp2-F4’, ‘F4-C4’, ‘C4-P4’, ‘P4-O2’, ‘Fp2-F8’, ‘F8-T4’, ‘T4-T6’, ‘T6-O2’, ‘Fz-Cz’, ‘Cz-Pz’, ‘F3-Fz’, ‘Fz-F4’, ‘C3-Cz’, ‘Cz-C4’, ‘P3-Pz’, ‘Pz-P4’. A neuropediatrician labeled each EEG recordings with the start time and the end time of each hypsarrhythmia stretches. Then, EEG signal stretches with hypsarrhythmia of the West Syndrome were labeled *Hips-WS*. EEG signal stretches with hypsarrhythmia from the Zika Virus Congenital Syndrome were labeled as *Hips-ZVCS*.

### Ethics statement

The protocols for this research were approved by Ethics Committee in Research of the University Hospital of the Federal University of Maranhão—CEP-HUUFMA, according to the attributions defined in the CNS Resolution no.466/2012 and CNS Operational Rule no. 001 of 2013 (Parecer Number: 2.111.125). All methods were performed in accordance with relevant guidelines and regulations. Informed consent was obtained from all parent and/or legal guardian participants included in the study.

### EEG signal preprocessing and feature extraction

The EEG signals selected from the described database were passed through a 6th order *Butterworth* low-pass filter with a cutoff frequency of 60 Hz to eliminate unwanted noise such as powerline noise. The definition of the 6th order *Butterworth* low-pass filter with a cutoff frequency of 60 Hz was done through researches in literature that specializes in other works that utilize EEG signal, aside from the realization of experimental tests to establish the order of the filter.

After filtering, the stretches labeled by the neuropediatric specialist as hypsarrhythmic were segmented into 1-s rectangular windows, with no overlap between them. Once there is no size of standard segment for the analysis of the EEG signal, the definition of the size of the segment was acquired through the simulation of the methodology proposed utilizing three different sizes: 1-s, 5-s and 10-s. This way, the 1-s segment was chosen based on accuracy of the results. Thus, we obtained 950 EEG signal segments of the (Hips-SCZV) class and 950 EEG signal segments of the (Hips-SW) class, totaling 1900 segments for analysis.

Non-stationary signals, like EEG signal, should be analysed by utilizing time domain and frequency domain techniques at the same time. This way, the application of the Continuous Wavelet Transform becomes adequate to reveal the activity profile of the EEG signal, once this technique provides good localization in both the time and frequency domains. Therefore, once the time-frequency behavior of the EEG signal is obtained, it is desirable to extract characteristics that take into consideration the variability of these two domains. This way, we utilize the time-frequency joint moments and entropy measurements to compose the relevant attributes fot the signal analysis.

EEG signal processing and feature extraction analysis are utilized in each of the 24 channels that compose the signal. This way, we carry out all the pre-processing procedures, that is, from the generation of the time-frequency distribution to the extraction of joint moments and entropy measurements while maintaining the spatial specificities of the EEG signal. Therefore, the intention is to maintain the relevant characteristics of the different regions of the brain, represented by each channel through the signals of these channels. So, after obtaining the joint moments and entropy measurements, these attributes are aggregated through attribute spatial integration indices.

### Time-frequency analysis based on continuous wavelet transform

The continuous wavelet transform calculates the correlation between the signal under consideration and the wavelet function $\psi (t)$. The similarity between the signal and the analyzed wavelet function, also called mother-wavelet function, is calculated separately at different time intervals, resulting in a two-dimensional representation^28,29^.

The continuous wavelet transform is defined as (1)^30^:

1 $$\begin{aligned} X_{TCW}(\tau ,s) = \frac{1}{\sqrt{|s|}}\int _{-\infty }^{\infty } x(t)\psi ^*(\frac{t-\tau }{s})dt. \end{aligned}$$

The signal transform $X_{TCW}(\tau ,s)$ is a function of the translation parameter $\tau$ and the scale parameter *s*. The mother-wavelet function $\psi ^*$ indicates that the complex conjugate is used in the case of a complex wavelet. The energy of the signal is normalized at each scale by dividing the wavelet coefficients by $1/\sqrt{|s|}$. This makes sure that the wavelets have the same energy at all scales^30^.

CWT was selected for this research as it includes time-based information. CWT produces a scalogram graph that consequently corresponds to the time-frequency spectrum graph. The scalogram is formed from the result of the signal correlation with the wavelet function in different scales throughout the time duration of the signal.

In observing the scalogram of an EEG signal, we are interested in spectral changes of energy throughout time and the application of the Continuous Wavelet Transform allows for the realization of these spectral analyses. The relation between scales and frequency in the CWT is not precise, however there is a counterpose between them, in the sense that low scale values correspond to high frequencies and vice versa. Still, it is necessary an approximation in order to relate the scales to the frequencies of the signal spectrum in analysis^29^.

The mapping between scales and frequencies can be done through (2):

2 $$\begin{aligned} F_j = \frac{F_c}{s_j}, \end{aligned}$$

where $F_c$ is the central frequency of the selected wavelet function and $F_j$ is the frequency corresponding to the $s_j$ scale. Therefore, for the approach used in the feature extraction phase, the continuous wavelet transform (CWT) was applied to generate an energy distribution in the time-frequency domain. From the application of the CWT, the energy variations in the different frequency subbands, included the principal subbands of the EEG signal [delta ($\delta$ 0–4 Hz), theta ($\theta$ 4–8 Hz), alpha ($\alpha$ 8–16 Hz), beta ($\beta$ 16–32 Hz), and gamma ($\gamma>$ 32 Hz)] over time could be determined, allowing for a multiresolution analysis of the signal. Three different analytic wavelet functions were used as the core of the Continuous Wavelet Transform. Then, the Morse, Morlet (Gabor), and Bump wavelet functions were evaluated in generating the time-frequency energy distributions of the Hips-ZVCS and Hips-WS signals.

All signal segments Hips-ZVCS and Hips-WS were decomposed by each of these wavelet functions. Then, we performed the signal reconstruction procedure for each of the mother wavelet functions and obtained the root mean squared error metric-RMSE. This metric calculates the square root of the mean squared difference between the values of the original signal and the values of the signal reconstructed by the Inverse Continuous Wavelet Transform, i.e., the squared error. This metric was calculated to verify which mother wavelet functions best characterizes the studied hypsarrhythmic EEG signals. The comparison of the average RMSE among the segments was performed among all wavelets. We used as a method of selection of the mother wavelet function the one that presented the lowest average RMSE value among the segments analyzed for all channels, both for the class Hips-ZVCS and the class Hips-WS. Thus, Morlet (Gabor) wavelet was selected as the mother wavelet function for feature extraction of the Hips-ZVCS and Hips-WS segments.

### Joint moment of the time-frequency distribution of EEG signal

The time-frequency distributions generated by time-frequency analysis techniques capture the behavior of the signal’s frequency variations over time. However, the direct treating of these distributions as attributes of the signal leads to a high computational effort and potentially introduces unrelated and undesirable features. In contrast, obtaining the moments in the low dimensionality time-frequency domain provides a method to capture the essential features of the signal in a much smaller data package. Using these moments significantly reduces the computational effort for feature extraction and comparison-a key benefit for real-time operation^31–33^.

The joint time-frequency moments of a nonstationary signal consist of a set of time-varying parameters that describe the signal spectrum as it evolves.

In theory, the joint moments of a signal can be obtained directly from (3):

3 $$\begin{aligned} \left\langle t^n \omega ^m \right\rangle = \int \int t^n \omega ^m \rho (t,\omega )dtd\omega . \end{aligned}$$

$\left\langle t^n \omega ^m \right\rangle$ is the joint time-frequency moment of a signal *x*(*t*). $t^n$ is the temporal moment of a signal *x*(*t*); $\omega ^m$ is the spectral moment of a signal *x*(*t*) and $\rho (t,\omega )$ is a joint time-frequency distribution.

In this paper, we extract the first four joint moments of the time-frequency distribution generated by the Continuous Wavelet Transform: joint mean—$\mu _{(t,f)}$, joint variance—$\sigma _{(t,f)}^2$, joint skewness—$\lambda _{(t,f)}$, and joint kurtosis—$\kappa _{(t,f)}$ for each class of signals Hips-ZVCS and Hips-WS. Thus, assuming that the time-frequency distributions of each class are generated by different probability density functions (pdf), these moments can be used to evaluate these pdf and be used to characterize each of the signal classes analyzed.

### Entropy measurement of of the time-frequency distribution of EEG signal

In recent years, the concept of entropy-derived measurements has been used in the analysis of physiological signals in studies assessing the complexity of biological systems. Entropy measurements are used to demonstrate the physiological complexity loss related to the appearance of a disease in the dynamics of various physiological systems, including cardiovascular, respiratory, and neurological systems^34,35^.

The analysis of the electroencephalogram signals from entropy measurements has proven to be appropriate because neuronal systems exhibit a non-linear or chaotic type of behavior. Thus, entropy is a way to quantify, in a statistical sense, the degree of uncertainty or randomness in the pattern, which is also roughly equivalent to the amount of information contained in the signal^36^.

Due to the property of entropy measuring the degree of disorder of a system, several forms of entropy have been developed. In this work, four of them were used: Shannon Entropy, Log Energy Entropy, Norm Entropy and Sure Entropy^37^ (for futher information, see Suplementary Information).

All entropy variations were obtained by taking the signal *X* and the expansion of its coefficients on some non-orthonormal basis $x_j$.

### Attribute spatial integration index generation

The EEG signals under analysis consisted of multiple channels (*ch*), in a total of 24. Thus, each segment of each of the classes (Hips-ZVCS) and (Hips-WS) constituted a matrix $\mathrm {M}_{Hips-ZVCS} = \mathrm {M}_{Hips-WS}|_{ch \times n}$, in which *n* is the number of samples per segment, given by multiplying the segment duration $T_{j}=1$ s by the sampling frequency $f_a$.

To apply the proposed methodology, we obtained the matrix sets $\mathcal {M}^j$ made up from *T*-th multichannel segment of the EEG signal, containing *ch* channels, of the pattern *j* to be recognized, i.e., the classes (Hips-ZVCS) and (Hips-WS), according to (4):

4 $$\begin{aligned} \mathcal {M}^j=\left\{ X_1^j, X_2^j,\ldots , X_T^j\right\} . \end{aligned}$$

*X* is a matrix containing the EEG signal samples of size $ch \times n$, in which *n* is the number of samples, in the time domain, per segment.

From the $\mathcal {M}^j$ set, the feature extraction step is performed. At this stage, the attribute spatial integration approach was considered to obtain the features of the signals that belong to the (Hips-ZVCS) and (Hips-WS) classes.

This approach is performed in such a way that features are extracted from each channel independently and then these extracted features are integrated to form a new unique feature set. Thus, the extracted features were combined through spatial averaging rather than concatenating them, to reduce the dimensionality problem of the resulting feature set. Thus, the attribute spatial integration index per segment is defined as (5):

5 $$\begin{aligned} F_a[T] = \frac{1}{N_{ch}}\sum _{i=1}^{N_{ch}}\frac{1}{N_a}\sum _{a=1}^{N_a}f_{ai}[T] \end{aligned}$$

in which $F_a[T]$ is the attribute spatial integration index of the feature *a* extracted from the *T*-th segment; $N_{ch}$ is the number of channels that make up the EEG signal segment; $N_a$ is the size of the feature vector and $f_{ai}[T]$ is the *a*-th coefficient of the feature vector of the *i*-th channel of the *T*-th analysis segment (for futher information, see suplementary material).

Thus, the Hips-ZVCS and Hips-WS classes are represented by the set $\mathcal {V}^j$ made up of the feature vector $V^j$, whose elements are the attribute spatial integration indices $F_{\mu _{(t, f)}}, F_{\sigma _{(t,f)}^2}, F_{\lambda _{(t,f)}}, F_{\kappa _{(t,f)}}, F_{E_{shannon}}$, $F_{E_{LogEnergy}}, F_{E_{Norm}}, F_{E_{Sure}}$ (6):

6 $$\begin{aligned} \mathcal {V}^j=\left\{ V_1^j, V_2^j,\ldots , V_T^j\right\} . \end{aligned}$$

### Machine learning classification methods

Machine learning techniques are extensively used as classifiers in medical decision support systems. These methods automatically process and recognize patterns in biological signals, including EEG signals, as healthy or unhealthy, using decision-making planes or surfaces that can discriminate between these situations. Eight main machine learning algorithms were evaluated in the discrimination process of the hypsarrhythmic EEG segments Hips-ZVCS and Hips-WS through the feature vector composed of joint moments and entropy measurements: Support Vector Machine (SVM)^38,39^; Discriminant Analysis (DA)^40,41^; K-nearest neighbour (k-NN)^42,43^, Gentle Boost Ensemble^44,45^, Decision Tree (DT)^46,47^; Logistic Regression (LR)^48,49^, Naive Bayes (NB)^50,51^, and Artificial Neural Networks (ANN)^52,53^. The hyperparameters are optimized using Bayesian optimization using expected improvement per second plus acquisition function^54,55^. The hyperparameters search range and optimized hyperparameters achieved can be seen in Supplementary Information.

### Performance measurements

The classification algorithms were evaluated from the metrics of accuracy, sensitivity, and specificity^1^. In addition to these metrics, a receiver operating characteristics curve (ROC) and an area under the curve (AUC), the Cohen’s kappa coefficient ($\kappa$), and the Matthews correlation coefficient (MCC) were analyzed.

The results presented by the classifiers indicate discrimination between the hypsarrhythmic EEG signal segments of ZVCS and WS. Thus, the analysis of these metrics allows us to determine how good and reliable the algorithm is at distinguishing between the two types of hypsarrhythmic segments from the extracted features.

Thus, the sensitivity metric reflects how efficient the classifier is in correctly identifying a true positive (TP); the specificity metric demonstrates how efficient the classifier is in correctly identifying the true negative (TN), and the accuracy indicates how efficient the method is in correctly diagnosing. The points on the ROC curve and the area under the curve (AUC) allow us to estimate how high the discriminating power of a classifier is.

The MCC and the $\kappa$ coefficient are correlation metrics, correspondence or reproducibility between the predicted classifications and true labels. Both coefficients vary in the range of [− 1, 1] and consider all four categories of the binary classification confusion matrix. MCC and $\kappa$ share various properties, where in both we can conclude that the result close to 1 indicates the case of very good prediction, meaning a strong correlation between the prediction and the true label; when the coefficients result in 0, it indicates a random prediction, that is, there is no correlation between the prediction and the true label; in the case of the coefficients resulting close to − 1, it indicates a total disagreement between the prediction and the true label^56^.

The equations for sensitivity, specificity, accuracy, MCC, and Cohen’s kappa coefficient ($\kappa$) can be found in the Supplementary Information.

The k-fold cross-validation method was used to derive and analyze these metrics in a credible and robust way regarding the classification efficiency. So, the whole dataset is randomly divided into k subsets with the same number of samples. The classifier model is tested with just one subset among the k subsets—for validation—and the other k − 1 subsets are assumed during the training of the classifier model. This procedure is replicated k times, so each k subset is used once for model validation. A second approach for validation of the models of the classifiers is the leave-one-patient-out cross validation. Being frequently used nowadays for validation of results of classification utilizing biometric signals, this method partitions k times the original set of samples of all patients in a way that the training partition is comprised of samples of patients that will not be contained in the test partition. The similarity of original samples from the same individual can be bigger than similarities between samples from different individuals. This way, the leave-one-patient-out cross-validation method implies that the characteristics of a testing set in each interaction will not be included in the training set, thereby ensuring the model’s reliability^57,58^.

Finally, the metric results from the k interactions are averaged to provide the performance of classifier models^59^.

## Results

In order to apply the methodology, we used 950 1-s multichannel segments of interictal EEG signals with hypsarrhythmia from ZVCS (Hips-ZVCS) and 950 1-s multichannel segments of interictal EEG signals with hypsarrhythmia from WS patients, totaling 1900 segments for verification.

As presented in this “Attribute spatial integration index generation”, the feature extraction step is performed in such a way that features are taken from each channel independently, and subsequently these extracted features are integrated to form a new unique set of features by calculating the attribute spatial integration index.

The first stage of feature extraction is the application of the Continuous Wavelet Transform to each of the 24 channels in each segment that make up the Hips-ZVCS and Hips-WS classes. Three different mother wavelet functions were analyzed as the kernel of the Continuous Wavelet Transform. Thus, the Morlet, Morlet (Gabor), and Bump mother wavelet functions were evaluated to select the wavelet that best describes the Hips-ZVCS and Hips-WS signals.

**Figure 2:**
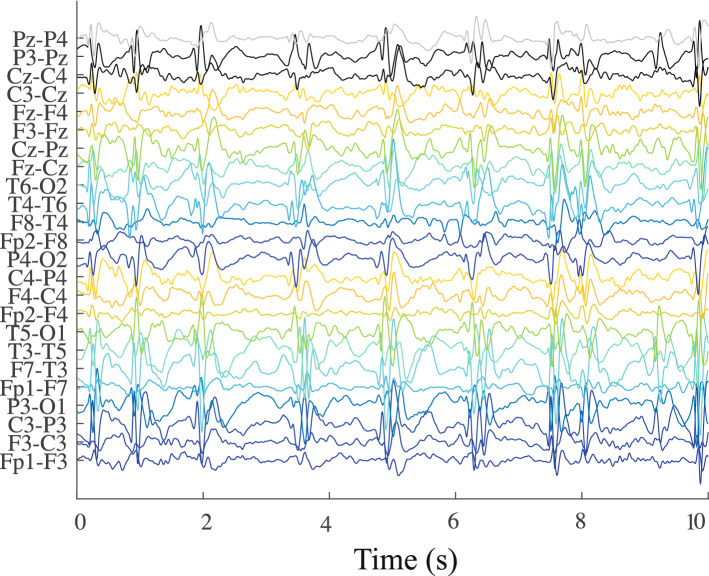
Hypsarrhythmic segments Zika virus congenital syndrome—Hips-ZVCS.

**Figure 3:**
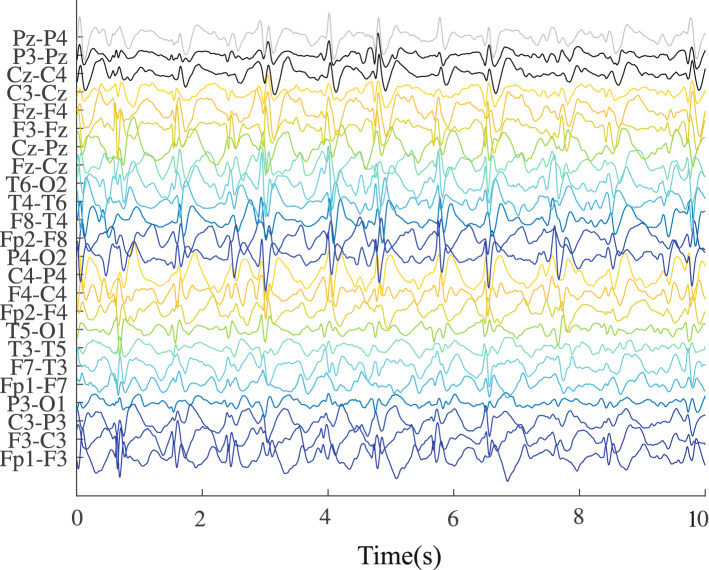
Hypsarrhythmic segments west syndrome—hips-WS.

Figure 2 represents 10 segments (Hips-ZVCS) without overlap and in Fig. 3 there are 10 segments (Hips-WS) without overlap, obtained from the database of EEG signals from the Ninar Support House.

Having defined the Morlet wavelet function as the kernel of the Continuous Wavelet Transform by the RMSE criterion (see Supplementary Information), the joint time-frequency moments: joint mean-$\mu _{(t,f)}$, joint variance-$\sigma _{(t,f)}^2$, joint skewness-$\lambda _{(t,f)}$ and joint kurtosis-$\kappa _{(t,f)}$ were obtained from the energy distributions generated from each channel by CWT using the Morlet wavelet function, as shown in “Attribute spatial integration index generation”.

Next, an attribute spatial integration index was generated for each of the joint moments per segment, according to 5. In this way, the indices $F_{\mu _{(t,f)}}[T]$, $F_{\sigma _{(t,f)}^2}[T]$, $F_{\lambda _{(t,f)}}[T]$ and $F_{\kappa _{(t,f)}}[T]$ were obtained for the *T*-th segment composing each of the Hips-ZVCS and Hips-WS classes.

Entropy measurements were also extracted from the time-frequency distributions of each channel generated by CWT, according to procedures explained in “Attribute spatial integration index generation”. Four types of entropy were obtained: Shannon Entropy, Log Energy Entropy, Sure Entropy, and Norm Entropy. Each of these entropy measurements constitutes a feature that were also aggregated using the Eq. (5). Thus, the following per-segment indices were obtained for the Hips-ZVCS and Hips-WS classes: $F_{E_{Shannon}}[T]$, $F_{E_{LogEnergy}}[T]$, $F_{E_{Norm}}[T]$ and $F_{E_{Sure}}[T]$.

**Figure 4:**
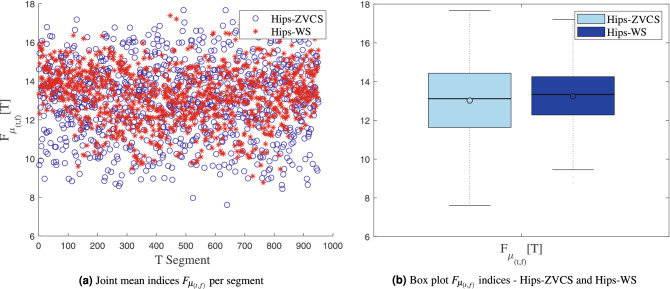
Distribution of the the joint time-frequency moment indices $F_{\mu _{(t,f)}}[T]$.

**Figure 5:**
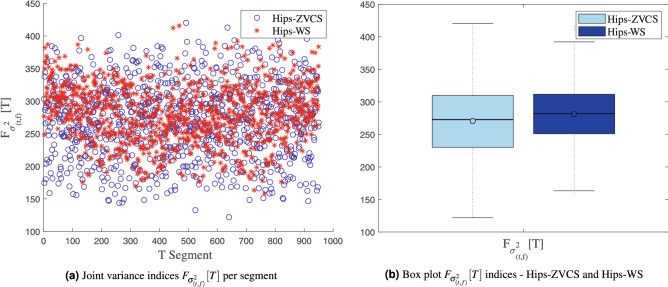
Distribution of the the joint time-frequency moment indices $F_{\sigma _{(t,f)}^2}[T]$.

**Figure 6:**
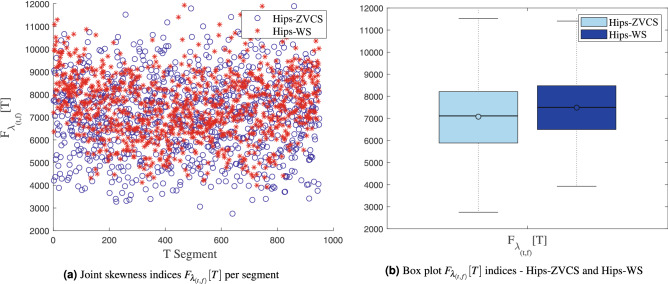
Distribution of the the joint time-frequency moment indices $F_{\lambda _{(t,f)}}[T]$.

**Figure 7:**
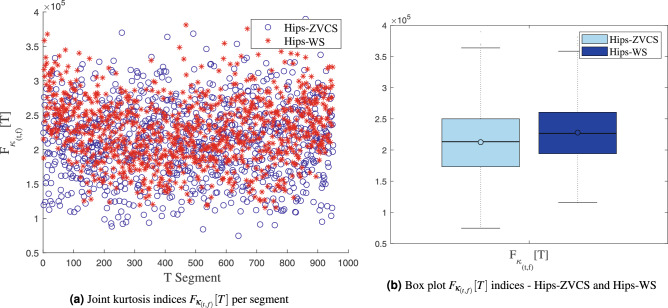
Distribution of the the joint time-frequency moment indices $F_{\kappa _{(t,f)}}[T]$.

The distribution of the the joint time-frequency moment indices $F_{\mu _{(t,f)}}[T]$, $F_{\sigma _{(t,f)}^2}[T]$, $F_{\lambda _{(t,f)}}[T]$ and $F_{\kappa _{(t,f)}}[T]$ of the Hips-ZVCS and Hips-WS classes is presented in Figs. 4, 5, 6, and 7, respectively .

**Figure 8:**
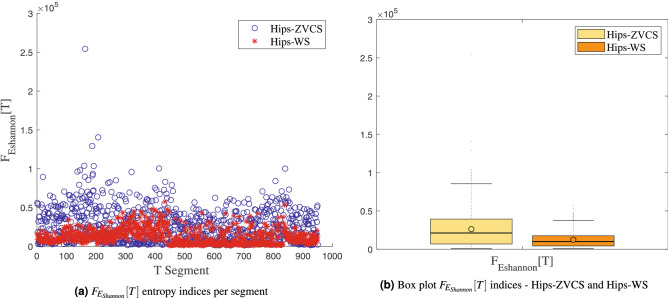
Distribution of the $F_{E_{Shannon}}[T]$ entropy measurement indices.

**Figure 9:**
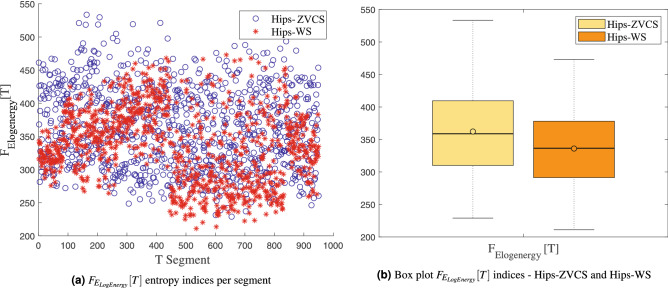
Distribution of the $F_{E_{LogEnergy}}[T]$ entropy measurement indices.

**Figure 10:**
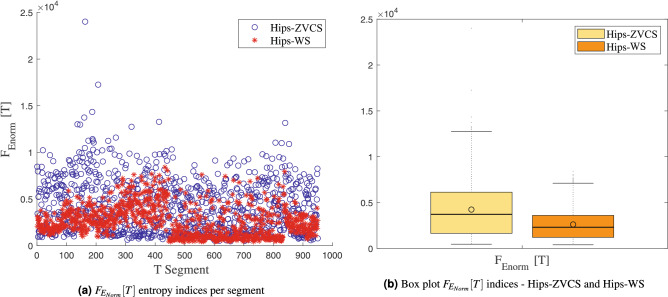
Distribution of the $F_{E_{Norm}}[T]$ entropy measurement indices.

**Figure 11:**
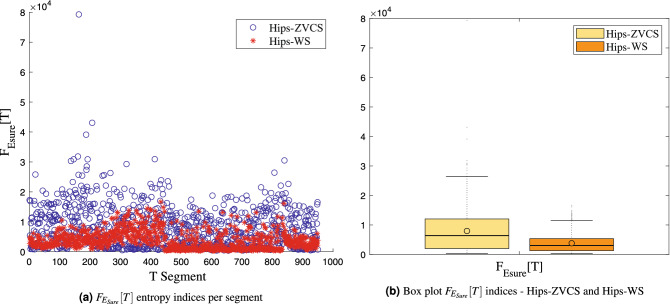
Distribution of the $F_{E_{Sure}}[T]$ entropy measurement indices.

The distribution of the indices $F_{E_{Shannon}}[T]$, $F_{E_{LogEnergy}}[T]$, $F_{E_{Norm}}[T]$ and $F_{E_{Sure}}[T]$ of the Hips-ZVCS and Hips-WS classes, respectively, is presented in Figs. 8, 9, 10, and 11.

The indices $F_{\mu _{(t,f)}}$, $F_{\sigma _{(t,f)}^2}$, $F_{\lambda _{(t,f)}}$, $F_{\kappa _{(t,f)}}$, $F_{E_{Shannon}}$, $F_{E_{LogEnergy}}$, $F_{E_{Norm}}$, and $F_{E_{Sure}}$ make up the feature vector that defines the Hips-ZVCS and Hips-WS classes. This gives an 8-dimensional feature vector.

For the verification of the statistical significance of indices of joint moments and the indices of entropy measurements, we did two hypothesis tests. In the first test, done with Kolmogorov–Smirnov, it was verified the null hypothesis that the data come from a normal distribution. In this test, the result is 1 (h = 1) if the test rejects the null hypothesis at a level of significance (p-value) of 5%. The second test was the Mann-Whitney test. In this test, we assessed the null hypothesis that the data come from continuous distribution samples with averages equal to the level of significance (p-value) of 5%. In this test, in case the null hypothesis is rejected, the result will be equal to (h = 1). The results obtained for the statistical significance of all indices in both tests are presented in Table 1.

We performed the two-by-two combination of the joint moment index versus joint moment index, entropy measurement index versus entropy measurement index, and joint moment index versus entropy measurement index. This was made to check the possibility that one of these combinations allows for separability between the Hips-ZVCS and Hips-WS segments in a lower dimension. The t-SNE algorithm was used to get an idea of how the feature vector consisting of the indices joint moment and entropy measure indices relate the Hips-ZVCS and Hips-WS classes. t-SNE is a dimensionality reduction algorithm whose goal is to enable the visualization of high-dimensional data.

**Table 1:** Statistical significance of indices of joint moments and entropy measurements.

Hypothesis test	Kolmogorov–Smirnov test		Mann–Whitney test	
	*h*	*p*-value	*h*	*p*-value
$F_{\mu _{(t,f)}}$	1	1.5333E−06	1	2.530E−02
$F_{\sigma _{(t,f)}^2}$	1	1.9566E−06	1	6.2513E−05
$F_{\lambda _{(t,f)}}$	1	2.6210E−07	1	1.7846E−07
$F_{\kappa _{(t,f)}}$	1	9.1151E−08	1	1.8037E−09
$F_{E_{LogEnergy}}$	1	9.4913E−15	1	6.2568E−18
$F_{E_{Norm}}$	1	3.8451E−35	1	1.1590E−36
$F_{E_{Shannon}}$	1	1.7596E−47	1	2.3732E−47
$F_{E_{Sure}}$	1	3.7669E−45	1	1.0592E−45

The idea of the algorithm is to embed high-dimensional points in lower dimensions in a way that respects the similarities between points. Near points in high dimensional space correspond to near points embedded in low dimension, and far points in high dimensional space correspond to far points embedded in low dimension^60,61^.

**Figure 12:**
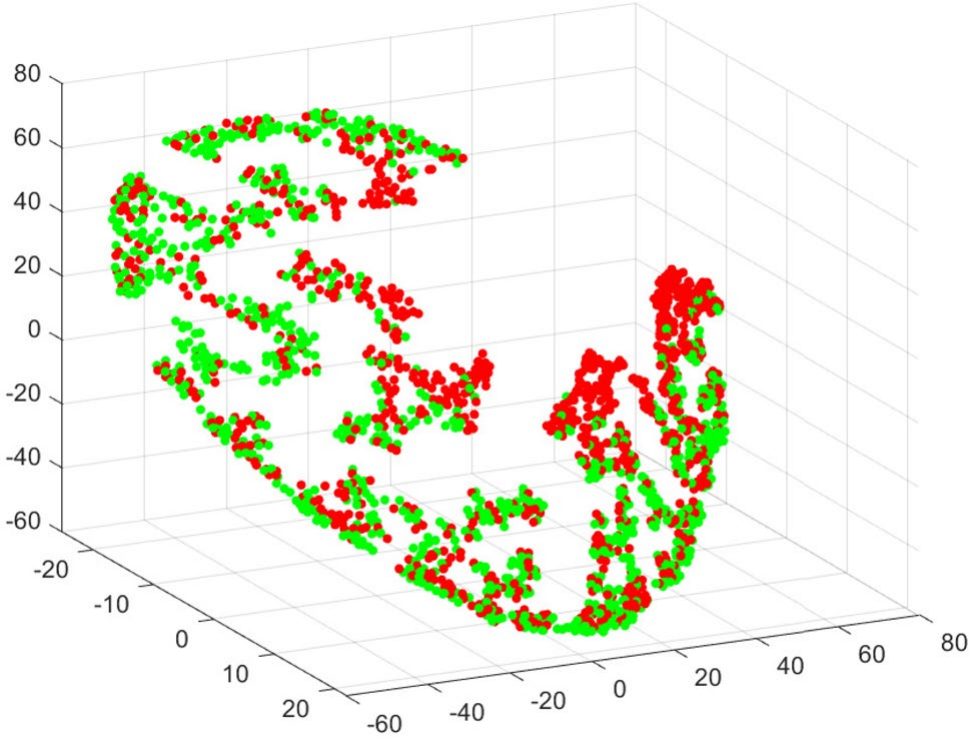
Visualization of Hips-ZVCS and Hips-WS classes by the t-SNE algorithm.

Figure 12 shows the visualization in three-dimensional space of the 8-dimensional feature vector that describes the Hips-ZVCS and Hips-WS classes using the t-SNE algorithm. In Fig. 12, the red points in 3D space represent the feature vectors of the Hips-ZVCS class, and the green points represent the feature vectors of the Hips-WS class.

Thus, we analyzed the performance of different machine learning methods for Hips-ZVCS and Hips-WS EEG signal classification: SVM, Discriminant Analysis, K-NN, Gentle Boost Ensemble, Decision Tree, Logistic Regression, Naive Bayes, and ANN. We selected the hyperparameters’values of the classifiers used based on the Bayesian optimization. These values were specified in the “Machine learning classification methods”. We used the k-fold cross-validation method and leave-one-patient-out cross validation method (Kfold = 5) for all two-by-two combinations of the indices.

In addition to the two-by-two combinations of the indices, we trained the classifiers using a vector with eight features, composed of four joint moment indices and four entropy measurement indices. This simulation was performed to verify whether the use of all indices increased the performance of the classifier.

After all the training, we observed that the classification results using the feature vector with all the indices obtained the best results in both validation methods. Thus, we present here only the results of the 8-dimension feature vector from the segments Hips-ZVCS and Hips-WS segments of the EEG signal. The average results of accuracy, sensitivity, specificity, precision, area under curve (AUC) of the ROC curve, Cohen’s kappa coefficient ($\kappa$) and Matthews correlation coefficient (MCC) obtained from the leave-one-patient-out cross validation and K-fold cross-validation for SVM, Discriminant Analysis, K-NN, Gentle Boost Ensemble, Decision Tree, Logistic Regression, Naive Bayes, and ANN are presented, respectively, in Tables 2 and 3. To implement leave-one-patient-out cross validation, for each iteration, the whole features per segment of one patient of each class was left out of the training set, which comprised the test set. We checked the statistical significance of the classification results obtained by calculating the assessment of classification performance at the chance level based on the number of data points available using a binomial cumulative distribution^62^. From this assessment, we verify that the accuracy of classifiers must exceeds the probabilistic chance level of 55.05% (for 950 observations, a 2-class, statistically significant at p < 0.001).

**Table 2:** Performance results for machine learning algorithms using leave-one-patient-out cross validation method.

Classifier	Accuracy (%)	Sensitivity (%)	Specificity (%)	Kappa	MCC	ROC_AUC
Decision tree	68.72	69.16	68.30	0.3744	0.3745	0.75
Discriminant analysis	72.15	84.40	66.33	0.4429	0.476	0.78
Gentle boost	73.52	72.10	75.12	0.4703	0.4713	0.82
k-nearest-neighbors	67.58	69.74	65.84	0.3516	0.3537	0.76
Logistic regression	74.79	79.19	71.55	0.4959	0.5016	0.85
Naive Bayes	63.70	68.29	60.95	0.274	0.283	0.65
Artificial neural network	78.08	85.55	73.21	0.5616	0.5745	0.89
Support vector machine	77.81	85.37	72.91	0.5562	0.5693	0.89

**Table 3:** Performance results for machine learning algorithms using k-fold cross validation method.

Classifier	Accuracy (%)	Sensitivity (%)	Specificity (%)	Kappa	MCC	ROC_AUC
Decision tree	74.42	71.05	77.79	0.4884	0.4895	0.80
Discriminant analysis	73.11	52.42	93.79	0.4621	0.5076	0.80
Gentle boost	79.26	76.95	81.58	0.5853	0.5859	0.88
k-nearest-neighbors	74.00	66.32	81.68	0.480	0.4858	0.83
Logistic regression	75.11	69.16	81.05	0.5021	0.5057	0.86
Naive Bayes	69.84	51.37	88.32	0.3968	0.4271	0.78
Artificial neural network	82.32	80.53	84.11	0.6463	0.6467	0.91
Support vector machine	81.05	76.42	85.68	0.6211	0.6237	0.91

## Discussion

In this paper, we present the results of the methodology proposed for characterization of interictal EEG signal segments with hypsarrhythmia in children with microcephaly caused by the Zika virus and interictal segments with hypsarrhythmia in children with West Syndrome. Hypsarrhythmia, a chaotic rhythm pattern present in interictal intervals in the EEG signal, was initially defined as a morphological marker in the EEG signal for diagnosis of West Syndrome. However, after the outbreak of children born with microcephaly due to the Zika virus infection in pregnant women, a chaotic pattern was also observed in these infants, which was characterized as the hypsarrhythmia pattern.

Due to limited knowledge about this new disease, some clinical studies have begun associating the congenital infection by the Zika Virus as the cause for West Syndrome (WS)^63,64^. Although the hypsarrhythmia found in the EEG of ZVCS is similar to the patterns more frequently described in malformations in the cortical development caused by other etiologies, in ZVCS, this one seems to be more complex and can cause continuous lesions, suggesting that ZVCS has morphological characteristics associated with the development of this pattern and, consequently, epilepsy in the first year of life^25,65,66^. Therefore, this direct association of symptoms seen in patients with Zika Virus Congenital Syndrome with WS makes it so that these patients receive the same consolidated treatment offered to patients diagnosed with WS. However, in many cases, there is no success in the response to these drugs in the control of the crises^64,65,67,68^. This way, the goals of the epilepsy treatment in the infancy are not very effective in the treatments currently offered to ZVCS patients.

Therefore, more in-depth studies on the specific characteristics of ZVCS, such as the analysis of the hypsarrhythmia pattern between these two syndromes carried out by this article, can instigate researches of new medications and treatments that may improve the quality of life of children with ZVCS, given that the results obtained by the developed method lead to a differentiation between the pattern in ZVCS and the ones seen in patients diagnosed with WS.

Then, based on the database obtained from the Ninar Support Home, stretches that showed the interictal pattern of hypsarrhythmia in ZVCS and hypsarrhythmia in WS are very similar visually. A certain periodicity of slow waves of high amplitude, mixed with peaks and discharges of sharp waves, stands out in these signals from the two classes of investigation.

The Morlet wavelet function was taken as the default function for the next steps of the feature extraction phase, as presented in the supplementary material.

Joint time-frequency moments and entropy measurements are the features extracted from the time-frequency energy distribution generated by CWT for each channel in each segment of the Hips-ZVCS and Hips-WS classes. As according to the proposed methodology, these techniques were applied to the time-frequency distributions of each channel and subsequently combined through the attribute-level spatial integration index, given by (5). Initially, the distribution along the segments of each index was analyzed independently, as a means of assessing the potential for discrimination between the Hips-ZVCS and Hips-WS classes of each index.

We notice some important characteristics of the distribution of these attributes when comparing the two classes. it is observed for the indices $F_{\mu _{(t,f)}}[T]$, $F_{\sigma _{(t,f)}^2}[T]$, $F_{\lambda _{(t,f)}}[T]$ and $F_{\kappa _{(t,f)}}[T]$ and for the indices $F_{E_{Shannon}}[T]$, $F_{E_{LogEnergy}}[T]$, $F_{E_{Norm}}[T]$ and $F_{E_{Sure}}[T]$ that the variability in the Hips-ZVCS class is larger relative to the variability of the Hips-WS class indices. Furthermore, it can be seen that there is no overlap between the medians of the two classes for all the indices analyzed, allowing for discrimination between the classes, although the difference between the medians of the two groups, for all the indices evaluated, is quite small.

According to the hypothesis tests done and presented in Table 1, it can be verified that all the indices obtained have statistical significance and reveal differences between the Hips-ZVCS e Hips-WS segments, therefore being relevant attributes to compose the feature vector. After the individual analysis of the features, a two-by-two combination of the obtained indices was performed. From the results obtained, we observed that a linear separation between the classes using any of the two-by-two combinations between the features is not possible.

Then, finalizing the analysis of the obtained indices, the t-SNE algorithm was used to visualize the Hips-ZVCS and Hips-WS classes represented by the 8-dimensional feature vector $V^j$ (Dimension = No. of spatial integration indices) in a low-dimensional space. As observed in Fig. 12, even with the classes represented by an 8-dimensional vector, a linear separation of the classes is still not possible, indicating that non-linear discrimination methods can assist in the classification between the Hips-ZVCS and Hips-WS segments.

The role of the classifiers in the proposed methodology is to reveal the contrast of the morphology of the hypsarrhythmia pattern of the EEG signal that presents in two childhood epileptic syndromes through the extracted features. The analysis of the metrics obtained by each classifier is crucial to determine the machine learning that best assists health professionals in the diagnosis and treatment of these patients.

To better understand the meaning of the metrics obtained through the results of the classifiers, we define in the context of the proposed study each of the terms that make up the calculation of these metrics, as follows:

1. Classes:class 1 = segments with hips-zvcs; class 2 = segments without hips-zvcs (hips-ws).
2. Metrics terms: True Positive (TP)—segments observed with hips-zvcs indicated as segments with hips-zvcs in the classification result; False Positive (FP)—observed segments without hips-zvcs (hips-ws) indicated as segments with hips-zvcs in the classification result; True Negative (TN)—observed segments without hips-zvcs (hips-ws) indicated as segments without hips-zvcs (hips-ws) in the classification result; False Negative (FN)—segments observed with hips-zvcs indicated as segments without hips-zvcs (hips-ws) in the classification result.

Thus, the numerical sensitivity values represent the likelihood of the classifier identifying hypsarrhythmic segments that actually belong to ZVCS. The higher this numeric sensitivity value is, less probable the classifier will return a false-positive result on further testing. Thus, a classifier that has high sensitivity tends to identify all possible hips-zvcs segments without discarding any cases. Thus, the classifier with high sensitivity allows for the trace-ability of segments with hips-zvcs.

Regarding specificity, the numerical result presented by the classifier represents the probability of identifying a segment without hips-zvcs, that is, a segment with hips-ws, as a true-negative. Thus, a high specificity of the classifier guarantees the trace-ability of hips-ws segments.

The numerical value of the accuracy allows us to conclude how well the classifier correctly identifies the TPs and TNs in the totality of the segments presented for diagnosis.

Therefore, a good classifier is one that has sensitivity, specificity, and accuracy with high numerical values simultaneously. We estimate chance level classification through the comparison between two validation methods: k-fold cross validation method (k-foldCV) and leave-one-patient out cross validation method (LOPOCV). The first method is vastly used in previous researches that analyze the EEG signal; while the second has been gaining attention because it is closer to clinical situations and because it diminishes an excessively optimistic evaluation of the classifiers results.

Thus, from Table 3, it can be seen that the Neural Network classifier presented the best results, simultaneously, for the metrics evaluated when k-foldCV method was applied. The neural network presented for this method 82.32% of accuracy, 80.53% of sensitivity, and 84.11% of specificity, all numerical values above 80%, without great disproportions among the metrics. These results presented by the Neural Network contrast with the results obtained for the Discriminant Analysis and Naive Bayes learning machines. For these two classifiers, a disproportion between sensitivity and specificity metrics is observed. Discriminant Analysis and Naive Bayes resulted in 52.42% and 51.37% sensitivity, respectively, and 93.79% and 88.32% specificity, respectively. Thus, these algorithms do not have a desirable response, since they are good at identifying hips-ws segments, but misclassify many hips-zvcs segments.

When we analyze the results of the classifiers using the LOPOCV method through Table 2, we verify that it has been a decrease in values of metrics. However, ANN still presented better results in relation to the other classifiers. The neural network presented for this method 78.08% accuracy, 85.55% sensitivity, and 73.21% specificity, maintaining a proportion among the metrics. We observed a variation among the metrics. For this method, it is observed that the disproportion between the sensitivity and specificity metrics in the classifier Discriminant Analysis that obtained 84.40% and 66.33% sensitivity and specificity, respectively.

It can be observed that for both cross-validation methods, the accuracy achieved by the ANN algorithm was also statistically significant, since it exceeded the threshold defined by the chance level of 55.05% at p < 0.001. Therefore, this metric ensures the validity of the proposed methodology.

The area under the ROC curve (AUC) is a way to measure the accuracy of the classifier, the closer the area is to unity, the better the accuracy of the classification. The commonly used classification result using the AUC is given by the following relationships: 0.9 < AUC < 1.0—Classification: Excellent; 0.8 < AUC < 0.9—Classification: Good; 0.7 < AUC < 0.8—Classification: Worthless; 0.6 < AUC < 0.7—Classification: Not good.

Therefore, through the results visualized in Table 3, the SVM and Neural Network algorithms present an excellent classification accuracy by the AUC criterion, presenting AUC = 0.91 in the k-foldCV method. Using the LOPOCV method, both classifiers acquired the value of AUC = 0,89; although it is a mildly lower value, it is still in the same performance range of the previous classification. Most of the classifiers were considered by this criterion to have good classification accuracy in both validation methods, with only the Naive Bayers classifier showing worthless accuracy in the leave-one-patient-out cross validation method.

The values obtained for the $\kappa$ index and the Mathews Correlation Coefficient were very similar between them for all the classifiers analyzed in both validation methods. There has been a decrease in values of the coefficients in the LOPOCV method in relation to k-foldCV. ANN was the classifier with the best correlation indices. The value of the $\kappa$ index acquired by ANN was 0.5616 and 0.6463 for LOPOCV and k-foldCV, respectively. On the other hand, the result of the MCC acquired by ANN was 0.5745 and 0.6467 for LOPOCV and k-foldCV, respectively. These results demonstrate that there is a moderate to substantial level of correlation between the real values and the values predicted by the classifier according to the scale presented in Ref.^69^.

In regards to the possibility of the age difference between both groups influencing the performance of the classification, we did not find any report of changes in the hypsarrhythmias pattern due to difference in age, in the interval we studied. In Ref.^70^, we found a study on the relation between the hypsarrhythmias pattern and age. In this article, the authors studied the duration of the hypsarrhythmias pattern for months and years in patients. The authors did not report changes in the first year of life.

Therefore, analyzing all these variables in both validation methods and the assessment of classification performance at the chance level, it is concluded that the Neural Network was well evaluated in both cases, thus being defined as the classifier in discriminating between the segments of the hypsarrhythmia pattern with ZVCS and WS, reliably revealing the existing differences between this pattern in the two syndromes. However, leave-one-person-out cross-validation method was the most trustworthy validation strategy because no same-patient segments were in both the training and in the test set at the same time. Thus, the metrics acquired by ANN using the leave-one-patient-out cross-validation method are considered the resulting metrics of this methodology.

## Conclusion

In this study, we presented a methodological strategy for the development of the analysis and differentiation, in the time- frequency domain, between the hypsarrhythmia pattern given in microcephaly caused by the Zika virus and the hypsarrhythmia pattern arising from West Syndrome using machine learning. We utilized a pipeline for the analysis of the EEG hypsarrhythmic signal of both syndromes, where we represented the pattern in analysis by an 8-dimensional feature vector composed of the indices of the joint moments and of the entropy measurements extracted from the time-frequency energy distribution obtained from the CTW. We calculate statistical significance of classification performance at the chance level based on binomial cumulative distribution. Eight classical types of machine learning algorithms had their performance evaluated in each of the validation methods through six metrics. The ANN classifier was able to achieve in the distinction between the Hips-ZVCS and Hips-WS segments 78.08% accuracy (higher than chance level of 55.05% at p < 0.001), 85.55% sensitivity, 73.21% specificity, AUC = 0.89, $\kappa$ = 0.5616, and MCC = 0.5745 for the leave-one-patient-out cross validation method.

## Supplementary Information

Supplementary Information.
