# Supplementary material for: Discrimination of secondary hypsarrhythmias to Zika virus congenital syndrome and west syndrome based on joint moments and entropy measurements
Source: Sci Rep. 2022 May 5;12:7389. doi: 10.1038/s41598-022-11395-2 (PMC9072419; doi:10.1038/s41598-022-11395-2)
Supplement: Supplementary file 1 — Supplementary Information. [file 41598_2022_11395_MOESM1_ESM.pdf]

Supplementary Material Document

Priscila Lima Rocha[1,*], Washington Luis Santos Silva[2], Patrícia da Silva Sousa[3], Antônio Augusto Moura da Silva[4], and Allan Kardec Barros[1]

[1]Department of Electrical Engineering, Laboratory for Biological Information Processing (PIB), Federal University of Maranhão (UFMA), São Luís-MA, CEP 65080-805, Brazil
[2]Department of ElectroElectronics, Federal Institute of Maranhão (IFMA), São Luís-MA, 65030-005,Brazil
[3]Department of Medicine, University Hospital of the Federal University of Maranhão, São Luís-MA, 65080-805, Brazil
[4]Department of Public Health, Federal University of Maranhão, São Luís-MA, 65080-805, Brasil
[*]priscila.rocha@ifma.edu.br

ABSTRACT

This supplementary document include Includes some theory subtopics, the results, and discussion about the choice of the mother-wavelet function for hypsarrhythmia EEG signal analysis.

Entropy measurement of of the time-frequency distribution of EEG signal

- Shannon Entropy

$$E(X) = -\sum_j x_j^2 log(x_j^2) \tag{1}$$

- Log Energy Entropy

$$E(X) = \sum_j log(x_j^2) \tag{2}$$

- Norm Entropy

$$E(X) = \sum_j |x_j|^p \tag{3}$$

where $p \geq 1$ in l^p norm

- SURE Entropy

$$E(X) = n - \sum_{j:|x_j|\leq p} 1 + \sum_i min(x_j^2, p) \tag{4}$$

in which $p \geq 0$-threshold.

Attribute spatial integration index generation

Then, initially, the Continuous Wavelet Transform is applied to each row of the X-matrix belonging to the set $\mathscr{M}^j$, in which each row of the X-matrix represents a channel of the EEG signal. Then, for the T-th $\mathscr{M}^j$ matrix, we obtain a set of time-frequency energy distribution matrices, represented by (5):

$$\mathscr{W}^j(t,f) = \left\{ \begin{bmatrix} W_1^{(t,f)} \\ W_2^{(t,f)} \\ \vdots \\ W_{ch}^{(t,f)} \end{bmatrix}_1, \begin{bmatrix} W_1^{(t,f)} \\ W_2^{(t,f)} \\ \vdots \\ W_{ch}^{(t,f)} \end{bmatrix}_2, \dots, \begin{bmatrix} W_1^{(t,f)} \\ W_2^{(t,f)} \\ \vdots \\ W_{ch}^{(t,f)} \end{bmatrix}_T \right\} \tag{5}$$

in which

$$W_{ch}^{(t,f)}(M\times n)=\begin{bmatrix}\omega_{s_1 1} & \omega_{s_1 2} & \cdots & \omega_{s_1 n}\\ \omega_{s_2 1} & \omega_{s_2 2} & \cdots & \omega_{s_2 n}\\ \vdots & \vdots & \ddots & \vdots\\ \omega_{s_M 1} & \omega_{s_M 2} & \cdots & \omega_{s_M n}\end{bmatrix}_T$$

is the time-frequency energy distribution matrix of the ch-th channel of the T-th segment. M is the number of scales of analysis of the Continuous Wavelet Transform, s_M is the M-th scale of the CWT and $\omega_{s_M n}$ is the n-th coefficient of CWT on the s_M scale.

Once the $\mathscr{W}^j(t,f)$ set is formed, four joint time-frequency moments are generated from the ch-th matrix $W_{ch}^{(t,f)}$ of the T-th segment: joint mean - $\mu_{(t,f)}$, joint variance - $\sigma^2_{(t,f)}$, joint skewness - $\lambda_{(t,f)}$, and joint kurtosis - $\kappa_{(t,f)}$. In this way, four sets of statistical metrics are obtained:

$$Md|^j_{\mu_{(t,f)}}=\left\{Md_1^j, Md_2^j, \dots, Md_T^j\right\} \tag{6}$$

$$Var|^j_{\sigma^2_{(t,f)}}=\left\{Var_1^j, Var_2^j, \dots, Var_T^j\right\} \tag{7}$$

$$Sk|^j_{\lambda_{(t,f)}}=\left\{Sk_1^j, Sk_2^j, \dots, Sk_T^j\right\} \tag{8}$$

$$Kurt|^j_{\kappa_{(t,f)}}=\left\{Kurt_1^j, Kurt_2^j, \dots, Kurt_T^j\right\} \tag{9}$$

in which

$$Md_T^j=\begin{bmatrix}\mu^1_{(t,f)}\\ \mu^2_{(t,f)}\\ \vdots\\ \mu^{ch}_{(t,f)}\end{bmatrix}_{ch\times 1} \tag{10}$$

$$Var_T^j=\begin{bmatrix}\sigma^{2^1}_{(t,f)}\\ \sigma^{2^2}_{(t,f)}\\ \vdots\\ \sigma^{2^{ch}}_{(t,f)}\end{bmatrix}_{ch\times 1} \tag{11}$$

$$Sk_T^j=\begin{bmatrix}\lambda^1_{(t,f)}\\ \lambda^2_{(t,f)}\\ \vdots\\ \lambda^{ch}_{(t,f)}\end{bmatrix}_{ch\times 1} \tag{12}$$

(13)

$$Kurt_T^j=\begin{bmatrix}\kappa^1_{(t,f)}\\ \kappa^2_{(t,f)}\\ \vdots\\ \kappa^{ch}_{(t,f)}\end{bmatrix}_{ch\times 1} \tag{14}$$

In addition to the joint moments (t,f), four types of entropy measurements were extracted from the $\mathscr{W}^j(t,f)$ array: Shannon Entropy (15), Log Energy Entropy (16), Sure Entropy (17) and Norm Entropy (18). For each matrix $W_{ch}^{(t,f)}(M\times n)$, an entropy value is obtained for the M-th row of the matrix. In this way, four sets of entropy measurements are generated:

$$\mathscr{E}^j_{shannon} = \left\{ \begin{bmatrix} E_1^{shannon} \\ E_2^{shannon} \\ \vdots \\ E_{ch}^{shannon} \end{bmatrix}_1, \begin{bmatrix} E_1^{shannon} \\ E_2^{shannon} \\ \vdots \\ E_{ch}^{shannon} \end{bmatrix}_2, \dots, \begin{bmatrix} E_1^{shannon} \\ E_2^{shannon} \\ \vdots \\ E_{ch}^{shannon} \end{bmatrix}_T \right\} \tag{15}$$

$$\mathscr{E}^j_{logenergy} = \left\{ \begin{bmatrix} E_1^{LogE} \\ E_2^{LogE} \\ \vdots \\ E_{ch}^{LogE} \end{bmatrix}_1, \begin{bmatrix} E_1^{LogE} \\ E_2^{LogE} \\ \vdots \\ E_{ch}^{LogE} \end{bmatrix}_2, \dots, \begin{bmatrix} E_1^{LogE} \\ E_2^{LogE} \\ \vdots \\ E_{ch}^{LogE} \end{bmatrix}_T \right\} \tag{16}$$

$$\mathscr{E}^j_{sure} = \left\{ \begin{bmatrix} E_1^{sure} \\ E_2^{sure} \\ \vdots \\ E_{ch}^{sure} \end{bmatrix}_1, \begin{bmatrix} E_1^{sure} \\ E_2^{sure} \\ \vdots \\ E_{ch}^{sure} \end{bmatrix}_2, \dots, \begin{bmatrix} E_1^{sure} \\ E_2^{sure} \\ \vdots \\ E_{ch}^{sure} \end{bmatrix}_T \right\} \tag{17}$$

$$\mathscr{E}^j_{norm} = \left\{ \begin{bmatrix} E_1^{norm} \\ E_2^{norm} \\ \vdots \\ E_{ch}^{norm} \end{bmatrix}_1, \begin{bmatrix} E_1^{norm} \\ E_2^{norm} \\ \vdots \\ E_{ch}^{norm} \end{bmatrix}_2, \dots, \begin{bmatrix} E_1^{norm} \\ E_2^{norm} \\ \vdots \\ E_{ch}^{norm} \end{bmatrix}_T \right\} \tag{18}$$

in which $E_{ch} = \begin{bmatrix} e_1 \\ e_2 \\ \vdots \\ e_M \end{bmatrix}$ is a vector containing the entropies extracted from M scales of the time-frequency energy distribution of each channel.

Therefore, the features extracted from the channels of each segment, given in the equations (6), (7), (8), (9), (15), (16), (17) and (18) are aggregated using the attribute spatial integration approach.

Performance measurements

Sensitivity, specificity, accuracy, MCC, and Cohen's kappa coefficient (κ) are defined respectively by (19), (20), (21), (22), and (23) :

$$Sens = \frac{TP}{TP+FN} \times 100 \tag{19}$$

$$Spec = \frac{TN}{TN+FP} \times 100 \tag{20}$$

$$Acc = \frac{TP+TN}{TP+TN+FP+FN} \times 100 \tag{21}$$

$$\kappa = \frac{2\cdot(TP\cdot TN - FP\cdot FN)}{(TP+FP)\cdot(FP+TN)+(TP+FN)\cdot(FN+TN)} \tag{22}$$

$$MCC = \frac{TP\cdot TN - FP\cdot FN}{\sqrt{(TP+FP)\cdot(TP+FN)\cdot(TN+FP)\cdot(TN+FN)}} \tag{23}$$

which FN is false negative and FP is false positive.

Results

We present in Figure S1, Figure S2, and Figure S3, respectively, the results of the time-frequency energy distribution using CWT for the 'Fz-Cz' channel for the Hips-ZVCS and Hips-WS classes using the Bump, Morlet (Gabor), and Morse wavelet function. The images (a) and (c) in Figure S1, Figure S2, and Figure S3 represent the time-frequency energy distribution for the Hips-ZVCS class signals. The images (b) and (d) in Figure S1, Figure S2, and Figure S3 represent the time-frequency energy distribution for the Hips-WS class signals. Images (a) and (b) are called scalograms, and in (c) and (d), we have the energy distribution in a three-dimensional plane. The colors represent the intensity of the signal energy: yellowish tones represent higher energy; blue tones, lower energy.

The average RMSE value across the analyzed segments for all channels in each of the classes was obtained. The lowest value of the RMSE metric was used as the criterion for choosing the mother wavelet function that best describes the hypsarrhythmic EEG signals. The comparison between the RMSE generated by the three mother wavelet functions for each channel of the signals for both the Hips-SCZV and Hips-SW classes is presented in Figure S4.

The image (a) in Figure S4 represents the bar graph with the RMSE result of each channel composing the EEG signal segment of the Hips-ZVCS class. The image (b) in Figure S4 represents the bar graph with the RMSE result of each channel composing the EEG signal segment of the Hips-WS class. Each channel composing the segments of each class was reconstructed by each of the mother wavelet functions used. Each bar contains the information regarding the three mother wavelet functions, and is represented by the different colors inside the bar.

The hyperparameters search range and optimized hyperparameters achieved for classification algorithms in Table S1.

Table S1. Classifiers' hyperparameters used during the simulations

Classifier	Hyperparameters Search Range	Optimized Hyperparameters
Decision Tree	Maximum number of splits: 1-1899<br>Split criterion: Gini's diversity index, Maximum deviance reduction	Maximum number od splits: 31<br>Split criterion: Gini's diversity index
Discriminant Analysis	Discriminant type: Linear, Quadratic, Diagonal Linear,<br>Diagonal Quadratic	Discriminant type: Quadratic
Logistic Regression	Solvers: 'newton-cg', 'sag', 'lbfgs'<br>Penalty: 'l1', 'l2', 'elasticnet'<br>C: 0.1-100	Solver: 'newton-cg'<br>Penalty: 'l2'<br>C = 100
Naive Bayes	Distribution names: Gaussian, Kernel<br>Kernel types: Gaussian, Box, Epanechnikov, Triangle	Distribution name: Kernel<br>Kernel type: Triangle
SVM	Multiclass method: One-vs-All, One-vc-One<br>Box constraint level: 0.001-1000<br>Kernel scale: 0.001-1000<br>Kernel function: Gaussian, Linear, Quadratic, Cubic	Multiclass method: One-vs-All<br>Box constraint level: 12.8883<br>Kernel scale: 1<br>Kernel function: Cubic
k-NN	Number of neighbors: 1-950<br>Distance metric: City block, Chebyshev, Correlation, Cosine, Euclidean,<br>Hamming, Jaccard, Mahalanobis, Minkowski(cubic), Spearman<br>Distance weight: Equal, Inverse, Squared inverse	Number neighbors: 29<br>Distance metric: City block<br>Distance weight: Squared inverse
Ensemble Method	Ensemble method: Bag, GentleBoost, LogitBoost, AdaBoost, RusBoost<br>Number of learners: 10-500<br>Learning rate: 0.001-1<br>Maximum number of splits: 1-1899<br>Number of predictors to sample: 1-8	Ensemble method: GentleBoost<br>Number of learners: 101<br>Learning rate: 0.001088<br>Maximum number of splits: 2<br>Number of predictors to sample: 8
ANN	Number of fully connected layers:1-3<br>Activation function: ReLU, Tanh, Sigmoid<br>Regularization strength (lambda): 5.2632e-9 - 52.6316<br>First layer size:1-300<br>Second layer size: 1-300<br>Third layer size: 1-300	Number of fully connected layers:1<br>Activation function: ReLU<br>Regularization strength (lambda):0<br>First layer size: 8

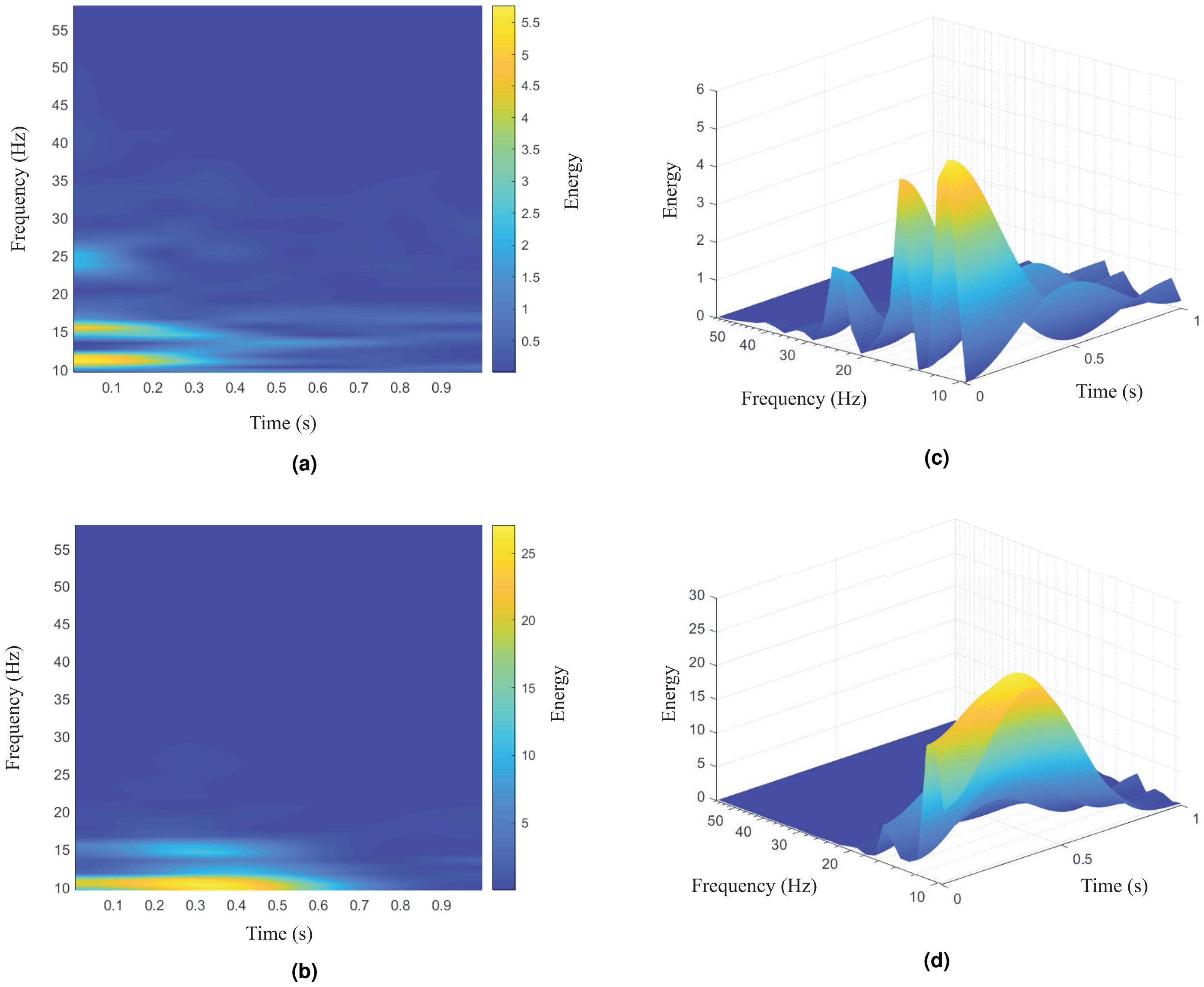

Figure S1. 'Fz-Cz' channel energy distribution for Hips-ZVCS and Hips-WS using Bump wavelet

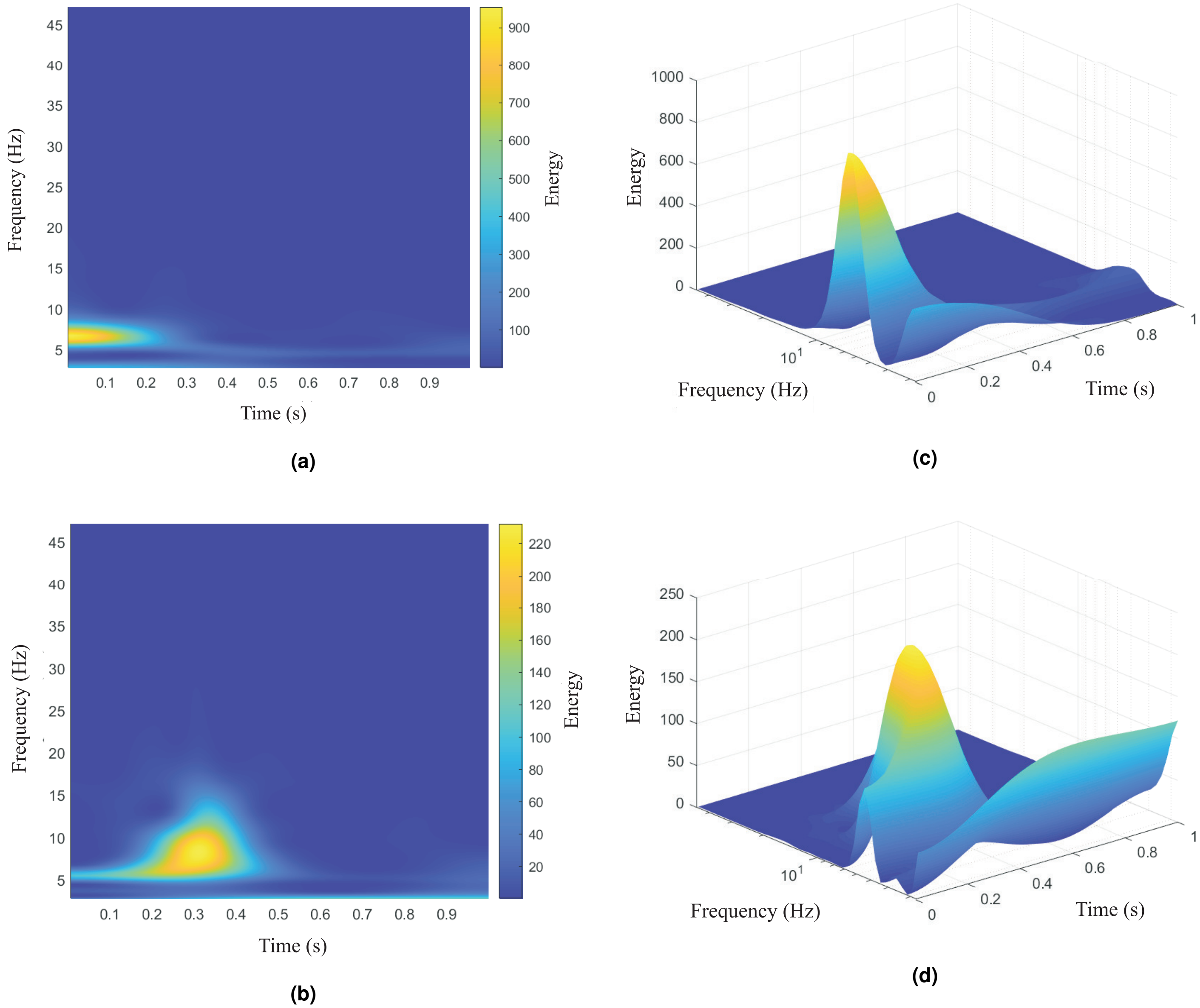

Figure S2. 'Fz-Cz' channel energy distribution for Hips-ZVCS and Hips-WS using Morlet (Gabor) wavelet

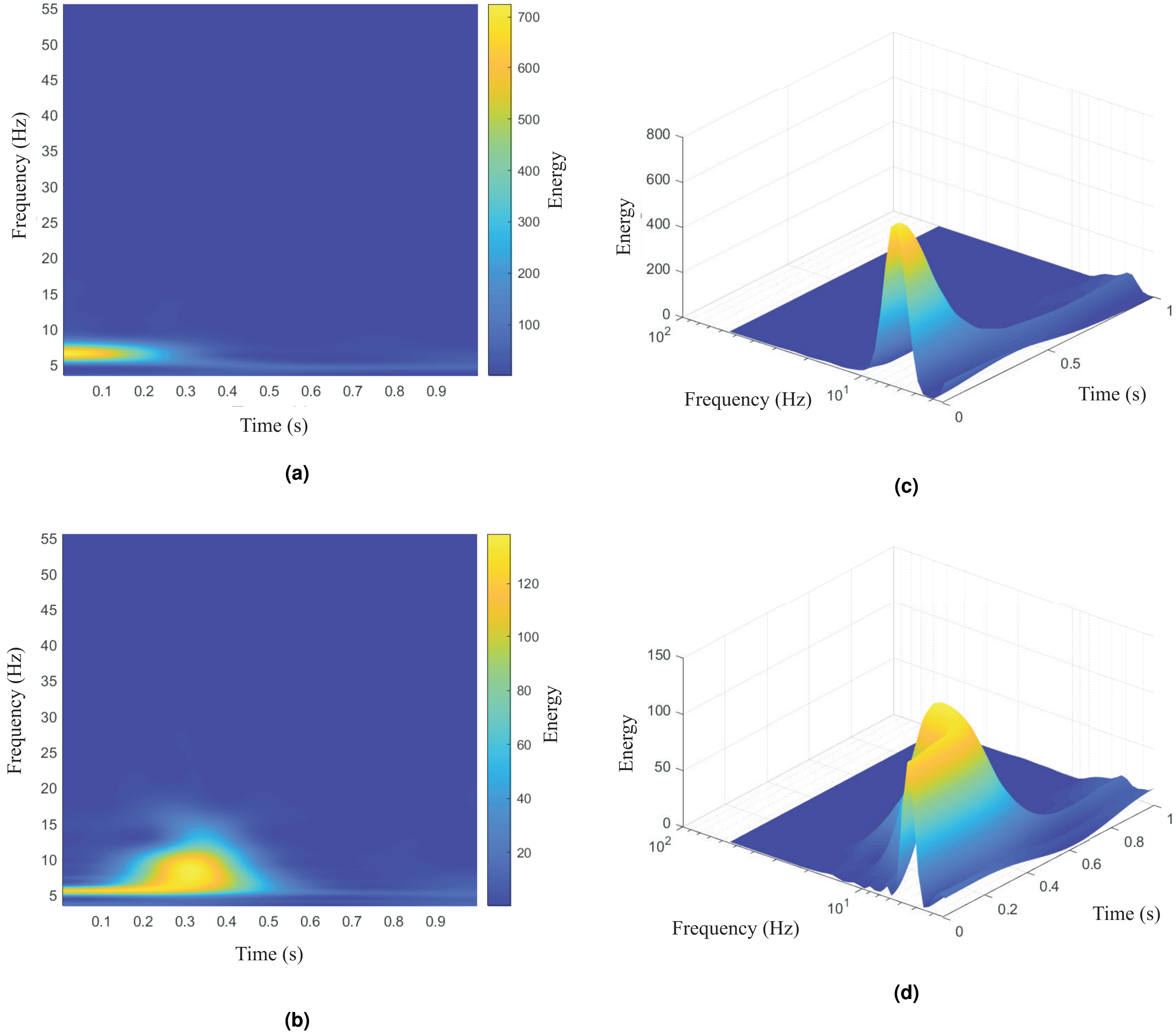

Figure S3. 'Fz-Cz' channel energy distribution for Hips-ZVCS and Hips-WS using Morse wavelet

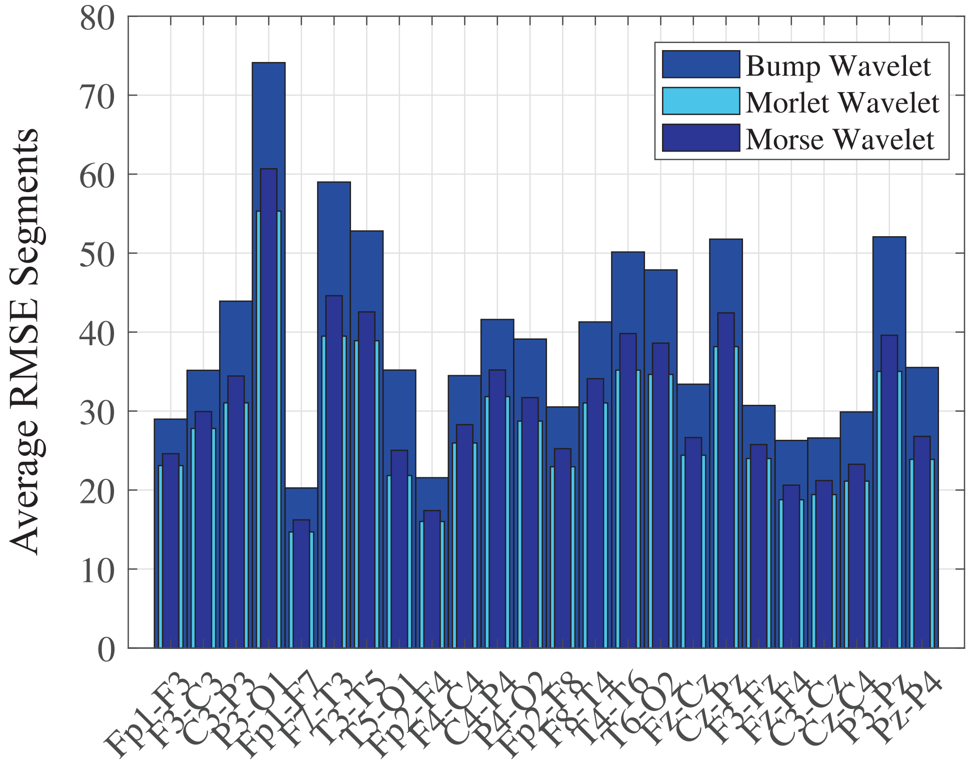

(a)

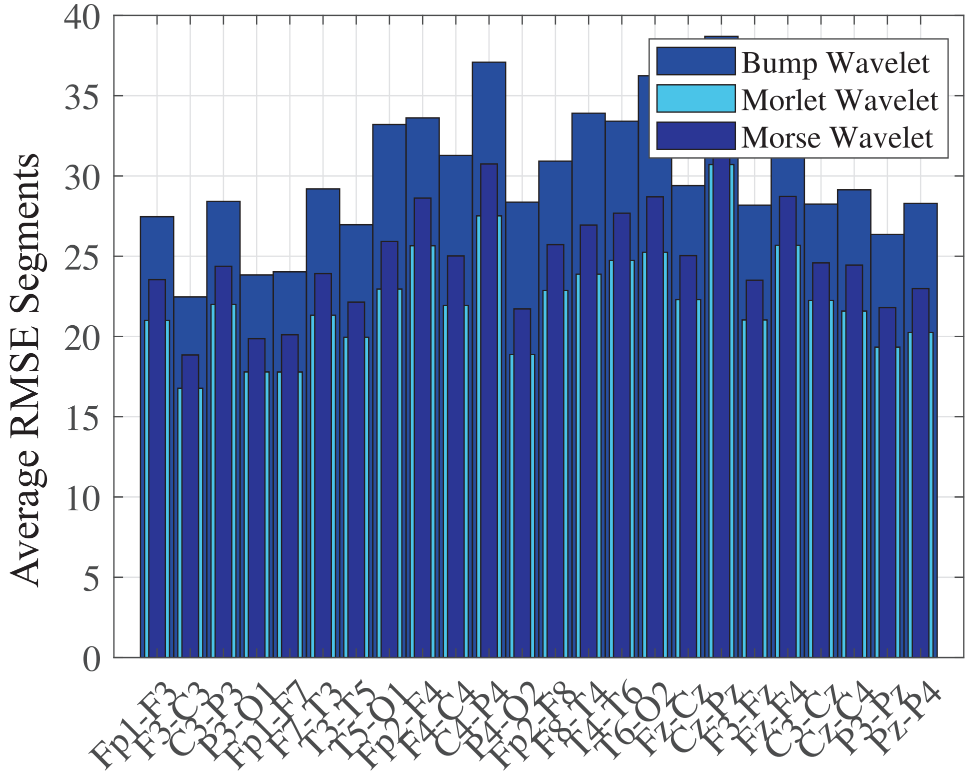

(b)

Figure S4. RMSE comparison between Bump, Morlet (Gabor) and Morse wavelet

1 Discussion

This complex of peaks and slow waves observed in the hypsarrhythmic signal demonstrates the multiple activity scales of the signal. Given its physiological nature, the EEG signal itself is a non-linear dynamic signal, in which it exhibits both time- and frequency-varying statistics, presenting a frequency content that varies over time. In this case of the hypsarrhythmic signal, the peak component of the waveform represents a short time scale event, and the slow wave component of the waveform represents a long time scale event.

Thus, the application of the Continuous Wavelet Transform becomes adequate to reveal the activity profile of the EEG signal of the Hips-ZVCS and Hips-WS classes simultaneously at various frequency scales and different time instants. It is observed in Figure S1(a) and (b), Figure S2 (a) and (b), Figure S3(a) and (b) that the highest energy content, represented by the more yellowish color in the graph is concentrated at low frequencies (0 - 15Hz) with a duration of a short time interval (0-0.4s). This behavior was verified in the other channels of the other segments for both classes.

As mentioned earlier, it is observed through these figures the change of activity in the complex of peaks and slow waves that make up these signals, where the high energy event observed reveals the peaks of the signal, as they are short duration events, while the slow waves present themselves throughout the time interval analyzed.

It can be seen in the scalogram and graph of the time-frequency energy distribution for both classes that the signal energy is concentrated mainly in the sub-bands δ, θ and α. These sub-bands were not analyzed in an individual way, but the information from all the sub-bands was relevant for the extraction of features.

The choice of the mother wavelet function that best describes the hypsarrhythmic signals of ZVCS and WS was performed from the RMSE (*root mean squared error*) generated by reconstructing the signal by inverse CWT with each of the mother wavelet functions. It was found that the Morlet wavelet function was the function that obtained the lowest average RMSE value across segments in the reconstruction of all channels in both classes. With the use of the Morlet function as the core of CWT, good results were also obtained in the analysis of EEG signals in the following works[1–3]. Therefore, the Morlet wavelet function was taken as the default function for the next steps of the feature extraction phase.

References

1. Sousa, G. C. L. d. *et al.* Identifcação do padrão de hipsarritmia em eletroencefalogramas: utilizando decomposição de sinais em pequenas ondas. (2019).

2. Prince, P. G. K. & Rani Hemamalini, R. Seizure detection using parameter estimation and morlet wavelet transform. In Krishna, P. V., Babu, M. R. & Ariwa, E. (eds.) *Global Trends in Information Systems and Software Applications*, 674–679 (Springer Berlin Heidelberg, Berlin, Heidelberg, 2012).

3. Bajaj, V. & Pachori, R. B. Automatic classification of sleep stages based on the time-frequency image of eeg signals. *Comput. Methods Programs Biomed.* **112**, 320–328, DOI: https://doi.org/10.1016/j.cmpb.2013.07.006 (2013).
